# Editorial: Advanced methodologies for studying function and therapeutic modulation of ion channels and transporter proteins

**DOI:** 10.3389/fphar.2026.1940237

**Published:** 2026-09-02

**Authors:** Jose Antonio Poveda, Timothy I. Webb, Takafumi Hara, Armando Alberola-Die

**Affiliations:** 1 IDiBE-Instituto de Investigación, Desarrollo e Innovación en Biotecnología Sanitaria de Elche, Universidad Miguel Hernández de Elche, Elche, Spain; 2 Independent Researcher, Brisbane, Australia; 3 Molecular and Cellular Physiology, Faculty of Pharmaceutical Sciences, Tokushima Bunri University, Tokushima, Japan; 4 Departamento de Fisiología, Genética y Microbiología, Universidad de Alicante, San Vicentedel Raspeig, Spain

**Keywords:** allosteric modulation, drug design, ion channels, single-molecule imaging, transporters

## 1 Introduction

Ion channels and transporter proteins are essential regulators of cellular homeostasis, and they are involved in virtually every aspect of physiology, including electrical signalling, ion and metabolite transport, and intracellular communication. Dysregulation of these membrane proteins contributes to the pathogenesis of numerous diseases, making them important therapeutic targets across diverse medical fields. Consequently, understanding the molecular mechanisms that govern their structure, function, and regulation remains a major focus of both basic and translational research.

In recent years, remarkable advances in experimental and computational methodologies have transformed the study of ion channels and transporters. New high-resolution structural techniques, molecular simulations, artificial intelligence (AI)-assisted approaches, advanced electrophysiological methods, and innovative pharmacological strategies provide unprecedented opportunities to investigate their conformational dynamics, elucidate molecular mechanisms, and accelerate the discovery of therapeutic modulators. These complementary approaches bridge the gap between structural insights and physiological function, facilitating the translation of fundamental discoveries into therapeutic applications.

This Research Topic, *Advanced Methodologies for Studying Function and Therapeutic Modulation of Ion Channels and Transporter Proteins*, was launched to showcase methodological advances that are reshaping research in this field.

The articles included in this Research Topic highlight complementary perspectives spanning structural biology, computational approaches, disease mechanisms, pharmacological modulation, and emerging therapeutic strategies. Collectively, they illustrate how innovative methodologies continue to deepen our understanding of ion channel and transporter biology while opening new avenues for therapeutic intervention.

## 2 Overview of the contributions

### 2.1 Review articles

The five review articles included here illustrate how recent advances in structural biology, the application of AI, disease-oriented ion channel research, and molecular trafficking studies are helping to reshape our understanding of ion channels and transporter proteins, providing new opportunities for therapeutic intervention.

Ashraf et al. review advances in antibody-enabled structural characterization of membrane proteins and discuss how AI-assisted antibody engineering is transforming this field. Because of their intrinsic instability and conformational heterogeneity, structural characterization of ion channels and transporters remains challenging. The authors describe how computational approaches facilitate antibody design, optimization, and structural prediction, accelerating the development of antibodies (and antibody fragments) targeting specific conformational states. They further highlight the emerging convergence of antibody engineering, protein-structure analysis, and machine learning as a powerful platform for antibody-enabled membrane structural biology.

Lamberti et al. summarize current evidence linking ion channels to chemotherapy-induced peripheral neuropathy (CIPN), a major dose-dependent toxicity of anticancer therapy. Ion channel dysfunction is identified as a key contributor to CIPN, unlocking new therapeutic avenues. Importantly, chemotherapy-induced changes in the regulation and sensitization of specific ion channels are shown to cause neuron hyperexcitability in dorsal root ganglion (DRGs) neurons. While experimental models, including mouse models and hIPSC-derived sensory neurons, have provided important mechanistic insights, significant translational gaps remain. AI-driven predictive models and an integrated approach (combining computational analyses with preclinical research, experimental animals, and human *in vitro* systems) may help bridge these gaps and facilitate the development of more effective therapies.

Haan et al. provide a comprehensive overview of ion channel translocation as a fundamental mechanism underlying plasticity. The review highlights common molecular mechanisms governing the exocytosis and endocytosis of ion channels during translocation. Regulators of specific endocytic processes such as FEME are also discussed, including endophilins (such as EndoA1) and Synaptotagmin-11. Additionally, they outline how dysfunction of these mechanisms may contribute to the pathophysiology of neurodegenerative disorders such as Parkinson’s and Alzheimer’s diseases. Hence, this important regulatory mechanism is a promising target for future therapeutic intervention.

Qiao et al. review recent advances in understanding the roles of transient receptor potential (TRP) channels in the pathogenesis of osteoarthritis (OA). TRP channels contribute to cartilage degradation and synovitis by modulating intracellular calcium signalling, inflammatory responses, and chondrocyte homeostasis (including apoptosis). They also play critical roles in pain signalling by mediating nociceptive signals in sensory neurons. During OA, pain sensitivity is enhanced *via* interactions with immune cells. Recent progress in developing TRP-targeted analgesic therapies is discussed, identifying TRPV1 modulators as especially promising for OA pain relief.

Xin et al. provide a comprehensive overview of the ER translocon protein Sec61, emphasizing its central role in membrane protein translocation, calcium homeostasis, and cellular physiology. The review further discusses the involvement of Sec61 channel dysfunction in genetic disorders and cancer, together with recent progress in the development of pharmacological Sec61-selective inhibitors. Currently, several inhibitors are being evaluated in clinical trials, and the importance of Sec61 as an emerging therapeutic target with broad clinical relevance is highlighted.

The technical, methodological, and conceptual advances described in these reviews should help inform future ion channel and transporter research and hopefully provide insights into the development of new therapeutic strategies.

### 2.2 Original articles

The three original articles complement these broader perspectives by providing new experimental evidence on channel gating, membrane trafficking, and pharmacological modulation.

Coll-Diez et al. investigate the structural basis of ion selectivity and permeation in the prokaryotic potassium channel KirBac1.1 after reconstitution in liposomes. Using patch clamp recordings and a fluorescence-based activity assay, they demonstrate that the selectivity filter adopts distinct conformations in the open state (at neutral pH) and closed state (at acidic pH), leading to marked changes in ion selectivity and permeation mechanisms. This work provides new mechanistic insights into channel gating and highlights the importance of structural approaches for understanding ion channel function.

Avalos-Hernandez et al. report on the effects of olive oil and oleic acid, its primary lipidic component, on the trafficking and functional expression of cyclic nucleotide-gated (CNG) channels, studied by patch-clamp recordings and fluorescence microscopy. These lipid components were demonstrated to improve membrane localization and functionality of mutant CNG channels, suggesting that they may act as lipid chaperones that promote proper membrane protein trafficking. These findings introduce a novel strategy for restoring membrane protein function through modulation of the cellular lipid environment.

Kappel et al. characterize the small-molecule inhibitor 4-chloro-2-(2-chlorophenoxy)acetamidobenzoic acid (CBA) as a pharmacological inhibitor of the proton-activated chloride channel TMEM206. Although inhibition of TMEM206 did not reduce acid-induced cell death in their *in vitro* colorectal cancer model, this study establishes CBA as a valuable pharmacological tool for investigating TMEM206 physiology. Moreover, it should facilitate the future development of more potent and selective TMEM206 inhibitors.

Together, these original studies demonstrate how complementary experimental, structural, and pharmacological methodologies can refine our understanding of ion channel and transporter structure and function, paving the way to develop new more potent and selective drugs able to modulate these key proteins.

## 3 Emerging trends and future perspectives

Our understanding of ion channels and transporters, including their structures, molecular mechanisms, and physiological regulation has advanced considerably over the past decades. Those intensive research efforts have contributed to the identification of disease mechanisms and the evaluation of ion channels and transporters as therapeutic targets, thereby strengthening the link between basic research and translational applications.

However, major challenges remain. Ion channels and transporters are regulated by complex conformational dynamics, subcellular trafficking, and interactions with diverse molecular partners, making it difficult to fully capture their behaviour using conventional experimental approaches alone. Overcoming these limitations requires a methodological innovation that integrates structural biology, physiology, pharmacology, and computational approaches.

As highlighted by several contributions in this Research Topic, AI-based methodologies are increasingly important in ion channel and transporter research. AI-assisted structural approaches offer powerful opportunities to analyse the dynamic conformational landscapes of channels and transporters, complementing experimental structural biology and expanding the scope of antibody-enabled structural investigations. AI-driven strategies may also accelerate the discovery and optimization of compounds that selectively modulate channel function, trafficking, or protein stability, strengthening the link between mechanistic studies and therapeutic development. In summary, the integration of experimental approaches with AI-based analyses will likely play a central role in overcoming many current technical limitations.

## 4 Concluding remarks

This Research Topic illustrates how methodological innovation continues to advance the study of ion channels and transporter proteins from multiple perspectives. By bringing together contributions spanning a range of methodologies, this Research Topic highlights both the breadth of the field and the expanding range of tools available to interrogate these important membrane proteins. Overcoming the challenge of translating fundamental mechanistic insights into clinical applications will require both continued experimental innovation and the effective integration of computational (especially AI-enabled) approaches.

Given the remarkable diversity of ion channels and transporters, continued methodological innovation will be essential for advancing our understanding of their biology and therapeutic potential. Nevertheless, we hope that this Research Topic will provide interested readers with a useful and stimulating overview of the methodological advances, emerging concepts, and future directions shaping this evolving field.

